# Photonic Biosensors: Detection, Analysis and Medical Diagnostics

**DOI:** 10.3390/bios12040238

**Published:** 2022-04-13

**Authors:** Donato Conteduca

**Affiliations:** Photonics Group, University of York, York YO10 5DD, UK; donato.conteduca@york.ac.uk

The necessity of personalised diagnoses and ad hoc treatments for individual patients is driving the outbreak of personalised nanomedicine in research and in clinical studies in the healthcare field. In this context, nanotechnologies are assuming a fundamental role due their ability to promptly diagnose a disease, its progression and aggressiveness, and their capability to define the most efficient drugs and treatments due to their continuous screening ability, making treatments less and less harmful for patients [1].

Together with the strong efforts to improve sensor performance, the demand to develop biosensors in low-cost and user-friendly technologies can no longer be neglected due to a forthcoming use of these sensing platforms in hospitals and clinics by healthcare workers.

Photonics biosensors have demonstrated their ability to combine high sensitivity, selectivity, and reliability together with real-time operation and strong integrability in cost-efficient solutions. Furthermore, the capability to integrate photonic biosensors with microfluidics and the compatibility of most of the photonic architectures and materials with electric readouts make them the most suitable candidates for the development of lab-on-a-chip systems to be integrated in point-of-care instruments [2].

The main goal of this Special Issue has been to address new technologies, materials, and configurations for photonic biosensors that can improve the sensitivity, accuracy, and precision of medical diagnostics. Novel biomedical studies have also been highlighted in this collection, i.e., for cardiovascular diseases and bacterial infections, which are now possible due to the technological progress and innovation that have been made in this field. Moreover, the strong demand for the integrability of the photonic biosensors in compact systems, which is playing a more fundamental role in many sensing applications, such as in wearable and implanted sensors, is becoming more evident.

The published articles, which are briefly described in the following section, cover these topics and make a strong impact in the biosensing field.

In [3], Adamopoulos et al. describe a novel strategy to create both micro and meso-scale microfluidic structures that were developed on multiple levels for integrated into monolithic photonic–electronic systems on a single chip. The strong advantage of silicon photonic sensors is their strong integrability with a printed circuit board (PCB) for the electrical readout and their compactness, allowing a large number of devices in a single photonic chip to undergo advanced multiplexing. However, the drawback is mainly related to the interface of the photonic sensors with the standard microfluidics. Standard packaging techniques usually suffer of this extreme compactness, which does not allow each sensor to be accessed and addressed without affecting the final cost of the chip. The authors solve this typical issue by creating a small microfluid interface that bonds to the silicon chip, accesses the single device, and uses micro-ring resonators as a sensing element as proof of concept. This microfluidic interfaces with another layer through vertical connections, where macro-scale tubes are directly connected. The realization of the multilevel microfluidics through the adoption of 3D printed transfer moulding and bonding can guarantee high scalability and cost efficiency.

In [4], Saha et al. developed a wearable device to measure blood circulation in human patients. The possibility of integrating the whole sensor in a watch-sized device represents a key technology for the next generation of biosensors because it allows for the continuous monitoring of health parameters in a fully non-invasive manner, something that could impact personalised medicine.

The authors developed a sensor based on laser Doppler flowmetry. The external sensor probe includes both an optical source and detector for the real-time measurement of blood perfusion by analysing the Doppler spectrum. The pilot study reported in this paper confirms the possibility of differentiating the cardiovascular parameters between smokers and non-smokers, findings that were confirmed by lower blood perfusion in the latter group and the presence of an embedded accelerometer that allows the blood perfusion levels to be discriminated from actual body movements.

A non-invasive sensing modality based on near-infrared (NIR) spectroscopy was also used by Heise et al. in [5] to measure blood glucose levels. The science-based calibration (SBC) method was applied by the authors and, in particular, tissue glucose concentrations were measured using the lip NIR-spectra of a type-1 diabetic patient recorded under modified oral glucose tolerance test (OGTT) conditions.

The reflection spectroscopy technique is preferred to transmission measurements because the latter requires thin skin folds or short-wave NIR spectroscopy to illuminate a fingertip or an earlobe. The main achievements of the above work are the demonstration that SBC calibration can be adopted for non-invasive studies and when selective measurements are needed. As confirmed by the authors, for further developments in this technology, more model parameters will be considered for in vivo spectroscopy, with physiology, sensor repositioning, temperature gradients, and blood flow changes being only a few of the main variables that are to be investigated in detail.

The necessity of continuous blood pressure screening using portable instruments and cost-efficient solutions is a recurring and pressing topic in healthcare technologies. Sun et al. in [6] adopted a convolutional neural network (CNN) based on the Hilbert–Huang Transform method, to predict the blood pressure risk level using photoplethysmography (PPG). The authors also proved that the derivatives of PPG carry important information on blood pressure, which could allow the combination of the ECG and PPG techniques to be replaced. Deep learning will be investigated in more detail in further studies in order to improve the accuracy of the measurements and the prediction of the blood pressure values related to specific physiological activities.

Rho et al. in [7] propose an optical cavity-based biosensor used for streptavidin and C-reactive protein (CRP) sensing. The optical cavity consists in two partially reflective and parallel surfaces separated by a thin gap, which affects the properties of the optical spectrum. Therefore, the gap is considered to be the sensing area that is functionalised with specific receptors. When the target analyte binds to the receptor, the changes in the optical properties provide changes in the optical intensities at two different wavelengths. The multi-wavelength system allows the cost of the system to be minimized by replacing the tunable laser and the spectrometer with low-cost laser diodes and a CMOS camera. The sensing modality is based on a differential detection method that maximizes the sensitivity, dynamic range, and fabrication tolerances. The authors have verified a limit-of-detection (LOD) of 377 pM for CRP by using a small sample volume of only 15 μL within 30 min.

Finally, in [8], Brunetti et al. modelled an optoelectronic biosensor to monitor bacterial biofilm evolution based on a multiparameter analysis with optical and electrical detection schemes. The sensor consists of a dual array comprising interdigitated micro- and nanoelectrodes in parallel. The optical response of the nanostructure is typical of a 1D grating supporting guided mode resonance (GMR). The resonant response allows the imaging of planktonic bacteria distributed in small colonies to be carried out at a higher spatial resolution, providing useful information on the modality of biofilm generation. In addition, the electrical response of both micro- and nanoelectrodes is necessary for the study of the metabolic state of the bacteria, which can be very powerful in the testing of the efficacy of antibiotics on the biofilm. The main advantage of this sensing configuration based on a multiparameter approach is the capability to detect and monitor a biofilm in real-time while also analysing its metabolic state and the evolution phase. Optoelectronic devices can make a strong impact in antimicrobial resistance studies.
